# Plant-Based Diets and Risk of Hospitalization with Respiratory Infection: Results from the Atherosclerosis Risk in Communities (ARIC) Study

**DOI:** 10.3390/nu15194265

**Published:** 2023-10-05

**Authors:** Karla N. Kendrick, Hyunju Kim, Casey M. Rebholz, Elizabeth Selvin, Lyn M. Steffen, Stephen P. Juraschek

**Affiliations:** 1 General Medicine, Beth Israel Deaconess Medical Center, Boston, MA 02215, USA; sjurasch@bidmc.harvard.edu; 2 Harvard Medical School, Boston, MA 02215, USA; 3 Beth Israel Lahey Health, Winchester Hospital Weight Management Center, Woburn, MA 01801, USA; 4 Johns Hopkins Bloomberg School of Public Health, Baltimore, MD 21287, USA; hkim25@jhu.edu (H.K.); crebhol1@jhu.edu (C.M.R.); eselvin@jhu.edu (E.S.); 5 Welch Center for Prevention, Epidemiology, and Clinical Research, Baltimore, MD 21287, USA; 6 University of Washington School of Public Health, Seattle, WA 98105, USA; 7 Johns Hopkins University School of Medicine, Baltimore, MD 21287, USA; 8 School of Public Health, University of Minnesota, Minneapolis, MN 55454, USA

**Keywords:** plant-based diet, healthy plant-based diet, unhealthy plant-based diet, vegetarian diet, infection, influenza, pneumonia, hospitalization

## Abstract

The benefits of plant-based diets may depend on the type of plant. To determine the associations of healthy and unhealthy plant-based diet types on risk of hospitalization with respiratory infections or any infection, we used dietary intake data reported in a food frequency questionnaire from the Atherosclerosis Risk in Communities Study to calculate a plant-based diet index (PDI), a healthy PDI (HPDI), and an unhealthy PDI (UPDI). Cox regression was used to calculate hazard ratios for the associations of the three plant-based diet indices with the risk of hospitalization with respiratory infections and any infection-related hospitalization. Comparing the highest to lowest quintiles, HPDI was associated with a lower risk of hospitalization with respiratory infections (HR 0.86, 95% CI: 0.75, 0.99), and a lower risk of hospitalization with any infections (HR 0.87, 95% CI: 0.78, 0.97). The PDI was associated with a lower risk of hospitalization with any infections (HR 0.86, 95% CI: 0.76, 0.96). Significant associations were not observed with the UPDI. Adults with a high PDI and HPDI had a lower risk of hospitalization with any infections, whereas adults with a high HPDI had lower risk of hospitalizations with respiratory infections.

## 1. Introduction

Plant-based diets are associated with a host of health benefits. Diets low in or without animal products are associated with lower risks of obesity, diabetes, cardiovascular disease, and cancer [1]. However, the association of plant-based diets on the risk of developing severe infection, particularly respiratory infection, is less clear. Some of the health benefits of plant-based diets are thought to be due to the modulation of inflammatory pathways and immune responses [2,3]. Several key micronutrients, such as vitamins A, C, and E, found in plant foods are essential for proper immune function, and deficiencies can lead to decreased activity of several immune cells such as macrocytic phagocytosis [3,4]. Therefore, it is possible that diets high in plant sources of food may contribute to a lower infection risk.

Plant-based diets have been classified into healthy and unhealthy based on the quality of plant foods and their associations with adverse health outcomes such as obesity, type 2 diabetes mellitus, cardiovascular disease, and cancer [5,6,7,8]. Unhealthy plant-based diets, high in refined grains, juice, sugar-sweetened beverages, sweets and desserts, and potatoes, have been associated with deleterious outcomes such as type 2 diabetes [5] and coronary artery disease [6]. This differential effect of a plant-based diet may extend to infectious outcomes as well.

The few studies that have examined associations of diet patterns and infectious outcomes have found associations of particular foods or diet types and risk of respiratory infection. Plant-based diets have been associated with a lower risk and severity of COVID-19 [9,10]. Low intake of fruits and vegetables has been associated with a higher risk of hospitalization with influenza [11]. In the Nurses’ Health Study, higher intake of oleic acid, found in vegetable oils, was associated with a lower risk of community-acquired pneumonia [12]. Among middle and older aged adults, plant-based diets derived using principal component analysis were associated with a lower risk of sepsis [13], but this association was attenuated after adjusting for sociodemographic, lifestyle, and clinical factors. These studies suggest that plant foods, particularly healthy plant foods, are beneficial in decreasing severe infection risk, particularly respiratory infections. However, studies exploring the differential associations of healthy and unhealthy plant-based diet patterns and the risk of hospitalization with respiratory infections, such as influenza and pneumonia, as well as infection overall, are lacking.

Our objective was to determine the associations of overall plant-based diets, as well as healthy and unhealthy plant-based diet types, on the risk of hospitalization with common respiratory infections and infections overall. We hypothesized that healthy plant-based diets and overall plant-based diets would be associated with a lower risk of hospitalization with common respiratory infections, such as pneumonia and influenza, while unhealthy plant-based diets would be associated with a higher risk of hospitalization with infections.

## 2. Methods

### 2.1. Study Design

The Atherosclerosis Risk in Communities Study is a community-based prospective cohort study, consisting of 15,792 middle-aged men and women between the ages of 45 and 64 at time of baseline enrollment from 1987 to 1989 [14]. Participants lived in the following four communities in the United States: Washington County, Maryland; Forsyth County, North Carolina; suburban Minneapolis, Minnesota; and Jackson, Mississippi. The institutional review board at each study site approved the protocol, and all participants provided informed consent.

Participants returned for follow-up visits multiple times from initial enrollment to the present day. Our analytic study sample consisted of 11,955 adults who completed dietary assessments at visit 3 (1993–1995) and met certain exclusion criteria. Participants with implausible total energy intake (<500 or >3500 kcal for women and <700 or >4500 kcal for men) were excluded, as well as those without complete covariate information (Figure S1). Participants who were hospitalized with influenza or pneumonia before visit 3 were excluded for the primary outcome. For the secondary outcome of hospitalization with any infection, participants with a prior hospitalization with infection were excluded.

### 2.2. Dietary Assessments

At visit 3, participants completed the 66-item, semi-quantitative Willett food frequency questionnaire [15], administered by trained interviewers, which was used to determine participants’ usual intake of foods and beverages. Participants indicated how often they consumed food and beverage items of a defined serving size. Nutrient and total energy intakes were derived by multiplying the intake amount of each food in the FFQ by its nutrient content.

### 2.3. Plant-Based Diet Score

We constructed an overall plant-based diet index (PDI), healthy plant-based diet index (HPDI), and a less healthy plant-based diet index (UPDI) based on responses to the FFQ, using methodology described previously [5,6,7,8]. These indices provided a nuanced characterization of plant food consumption. To create these indices, food items in the FFQ were categorized into 17 food groups, which were then classified into 3 food types (healthy plant foods, less healthy plant foods, and animal foods). Healthy plant foods consisted of whole grains, fruits, vegetables, nuts, legumes, coffee, and tea. Less healthy plant foods consisted of fruit juices, refined grains, potatoes, sugar-sweetened and artificially sweetened beverages, sweets, and desserts. Lastly, animal foods consisted of animal fat, dairy, eggs, fish or seafood, meat, and miscellaneous animal foods. We summed food items (servings/day) within the 17 food groups, and ranked participants by their energy-adjusted consumption of food groups into quintiles.

All diet indices scored plant foods according to their ranked consumption. For example, those in the highest quintile of fruit or potato consumption receive a score of 5, and those in the lowest quintile receive a score of 1. All diet indices reversed scored animal foods. For instance, those in the highest quintile of meat consumption received a score of 1, and those in the lowest quintile received a score of 5. For the PDI, those with the highest quintile of consumption of plant foods received the highest scores. For the HPDI, those with the highest quintile of consumption of healthy plant foods received the highest score for consumption of these foods. For the UPDI, those with the highest consumption of unhealthy plant foods received the highest scores. The PDI, HPDI, and UPDI had a possible range from 17 to 85. All scores were divided into quintiles for analysis. Consumption of the components of the diet indices, healthy plant foods, less healthy plant foods, and animal foods were also ranked into quintiles for analysis.

### 2.4. Outcome Assessment

Our primary outcome was hospitalization with respiratory infection, including influenza and pneumonia. Our secondary outcome was hospitalization with any infectious cause. Hospitalizations were identified through participant self-report during annual, as well as semi-annual (six months post annual interview) telephone interviews starting in 2012, and through active surveillance of hospital discharges in the communities in which participants lived. Diagnostic codes from hospital discharges were used to extract hospitalizations with infections, and specifically pneumonia and influenza. Codes of interest include those with pneumonia and influenza (ICD-10: J09-J18; ICD 9: 480–488), and those related to acute non-respiratory infections, such as cellulitis, urinary tract infections, pyelonephritis, meningitis, infectious colitis, bacteremia, and other viral, bacterial, and protozoal infections (ICD-10: A00-B99, G00-07, L00-008, N10, N30, N39.0, R78.81; ICD-9: 001–139, 320–324, 590, 595, 680–686, 790.7). Any codes within these ranges were used, given possible variation in physician selection of billing diagnoses. We included any hospitalization with the above codes, regardless of if it was the primary cause of hospitalization.

### 2.5. Covariates

Covariates included race/center (to account for the way the race groups are distributed across the centers), age, sex (male, female), education level (no high school diploma, high school diploma, some college and above), income level (≤$25,000, $25,000–49,999, ≥$50,000), alcohol intake (grams/week), current tobacco use (yes/no), and history of chronic respiratory disease (asthma, chronic obstructive pulmonary disease [COPD]), as determined by self-reported medical history. Body mass index (BMI) was obtained from height and weight measurements taken at visit 3. We used demographic information from visit 1 and visit 3. Characteristics such as sex, race/ethnicity, study site, education, and income were captured from visit 1. Race/center is defined as White adults in Washington County, Maryland; Black adults in Forsyth County, North Carolina; White adults in Forsyth County, North Carolina; White adults in Minneapolis, Minnesota; and Black adults in Jackson, Mississippi. Characteristics such as education level, income level, alcohol intake, tobacco use, body mass index, history of diabetes mellitus, and history of chronic respiratory disease (asthma, COPD) were captured from visit 3. Total energy intake was also included as a covariate to prevent confounding due to possible variation in the number of servings of food items in high or low caloric diets.

### 2.6. Statistical Analysis

Baseline characteristics of participants and food, vitamin, and mineral intake of participants were described by quintiles of the PDI. For participants meeting the criteria above, Cox regression was used to calculate hazard ratios and 95% confidence intervals to estimate the risk of hospitalization with pneumonia or influenza and then all infections based on diet types. The proportional hazards assumption was evaluated with a Schoenfeld residual plot, which supported proportionality. Length of follow-up time or time to first hospitalization was used as the time metric. Hospitalization surveillance proceeded through 31 December 2019 and annual follow-up through 8 January 2020. Participants without an event were censored at either the last date of annual follow-up or hospitalization. Three Cox proportional hazards models were used. The first model was adjusted for age, sex, race/center, and total energy intake. Model 2 further adjusted for potential confounders of the association of diet type and hospitalization with infections, including income, education, alcohol and tobacco use, and history of asthma or COPD. Lastly, model 3 further adjusted for BMI and diabetes as pre-specified potential mediators of the association of diet and severe infection. The median value within each quintile of the PDI, HPDI, UPDI and their components, healthy plant foods, less healthy plant foods, and animal foods, was used to test for a linear trend. We consider the main results to be estimates from model 2, without adjustment for mediating variables.

In addition to diet indices, we created models using the score components of the PDI simultaneously (quintiles of healthy plant foods, less healthy plant foods, and animal foods), and models using all 17 food groups simultaneously.

All analyses were performed in SAS 9.4 (Cary, NC, USA). Statistical significance was set at *p* < 0.05 and was not adjusted for multiple comparisons, given the interrelatedness of the outcomes.

## 3. Results

### 3.1. Participant Characteristics

Participant characteristics are described by quintiles of PDI (Table 1). A total of 59% of those in the highest quintile of the PDI were women, compared to 44.1% in the lowest quintile. Those in the highest quintile were more likely to be White and non-smokers. Average alcohol intake was also lower among those in the highest quintile compared to those in the lowest quintile (29 g/week vs. 68.5 g/week). Income and education were higher with higher quintiles of PDI. Diabetes and COPD diagnoses were less common in the highest quintiles of PDI, while history of asthma was similar across all quintiles of PDI.

### 3.2. Diet Indices

The PDI ranged from 18 to 72 points (Table 1). The HPDI ranged from 26 to 77 points (Table S1). The UPDI ranged from 20 to 76 points (Table S2). Those in the lowest quintiles of PDI and HPDI had higher BMIs and were more likely to currently use tobacco and have COPD and diabetes (Table 1 and Table S1). Conversely, across quintiles of UPDI, the distribution of White individuals and females was roughly the same (Table S2). Those in the lowest quintiles of UPDI were more likely to be college educated. Similarly, BMI and tobacco use were about the same across quintiles of UPDI, whereas alcohol intake was higher at higher quintiles of UPDI. COPD was similar across quintiles, and diabetes prevalence was lower at higher quintiles of UPDI.

With regard to nutritional characteristics, those in the highest quintiles of PDI and HPDI consumed an average of 4.7 to 5.6 servings of fruits and vegetables per day and 1.2 to 1.4 servings of red meat per day (Table 1 and Table S1). Those in the highest quintiles of PDI and HPDI also consumed more micronutrients such as vitamin C, vitamin A, folate, potassium, magnesium, and iron relative to those in lower quintiles of PDI and HPDI. In contrast, those in the highest quintiles of the UPDI consumed around 3 servings of fruits and vegetables per day and 1.6 servings of red meat per day. Micronutrient consumption varied across quintiles of UPDI in a non-linear fashion, with those in the highest and lowest quintiles having similar intakes of micronutrients, such as vitamin C, folate, iron, calcium, magnesium, and potassium.

### 3.3. Plant-Based Diets and Hospitalization with Respiratory Infections

During a median follow up of 22.4 years, there were 2522 hospitalizations with influenza or pneumonia. Incidence rates for respiratory infection hospitalizations were higher at lower quintiles of PDI and HPDI (Table S3, Figures S2 and S3). For UPDI, incidence rates followed in a U-shaped pattern, with incident respiratory infection being highest at the lowest and highest quintiles of UPDI, and lowest in the middle quintile (Table S3, Figure S4). Associations of diet index and risk of hospitalization with respiratory infection were strongest for HPDI (Table 2).

In all models for HPDI (Table 2), there was a significant trend (*p* < 0.001–0.037) of lower risk of respiratory infection with higher consumption of healthy plant foods. In the model adjusted for sociodemographic factors (age, race/center, education, income), health behaviors (tobacco and alcohol use), health conditions (asthma, COPD), and caloric intake, those in the highest quintile of HPDI had a 16% (HR 0.84 [95% CI: 0.73, 0.97]) lower risk of hospitalization with respiratory infections compared to those in the lowest quintile. A similar finding was observed in models further adjusted for potential mediating factors, such as diabetes and BMI, with those in the highest quintile of HPDI having a 14% (HR 0.86 [95% CI: 0.75, 0.99]) lower risk of hospitalization with respiratory infections compared to those in the lowest quintile. Significant associations with hospitalizations with respiratory infections were not seen with the PDI and UPDI.

### 3.4. Components of Diet Indices and Hospitalization with Respiratory Infections

Those in the highest quintiles of less healthy plant food consumption and animal food consumption had a significantly higher risk of hospitalization with respiratory infections, 24% (HR 1.24 [95% CI: 1.04, 1.48]) and 21% (HR 1.21 [95% CI: 1.01, 1.46]), respectively, compared to those in the lowest quintiles. However, the association for animal foods was no longer significant when we adjusted for BMI and diabetes (Table S4). There were no significant trends for consumption of healthy plant foods. In fully adjusted models, those in the highest quintile of healthy plant food consumption continued to have a significantly lower risk, 16% (HR 0.84 [95% CI: 0.71, 0.98]), of hospitalization with respiratory infections compared to those in the lowest quintile. With regards to food groups, higher intake of sugar-sweetened beverages was associated with higher risk of hospitalization with respiratory infections across all models (Table S5). Inconsistent trends were seen with other food groups and risk of hospitalization.

### 3.5. Plant-Based Diets and Hospitalization with Any Infection

During a median follow up time of 21.2 years, there were 4308 hospitalizations with any infection. Incidence rates with infectious hospitalizations were higher at lower quintiles of PDI and HPDI (Table S6). For UPDI, incidence rates followed in a “U-shaped” pattern, with incident infection being highest at the lowest and highest quintiles of UPDI, and lowest in the middle quintile. Associations of diet index and risk of hospitalization with any infection were strongest for PDI (Table 3).

In all models for PDI and HPDI, there was a significant trend (*p* < 0.001–0.006 and *p* < 0.001–0.009, respectively) of lower risk of infection with higher consumption of plant foods and healthy plant foods. In the model adjusted for sociodemographic factors, health behaviors, health conditions, and caloric intake, those in the highest quintile of PDI and HPDI had an 18% (HR 0.82 [95% CI: 0.74, 0.92]) and 16% (HR 0.84 [95% CI: 0.76, 0.94]) lower risk of hospitalization with infections, respectively, compared to those in the lowest quintiles. These findings were also observed in models further adjusted for potential mediating factors (diabetes and BMI), with those in the highest quintile of PDI and HPDI having a 15% (HR 0.85 [95% CI: 0.76, 0.96]) and 13% (HR 0.87 [95% CI: 0.78, 0.97) lower risk of hospitalization with infections, respectively, compared to those in the lowest quintiles. Statistically significant associations with hospitalization with any infection were not seen with the UPDI.

### 3.6. Components of Diet Indices and Hospitalization with Any Infection

When modeling components of the PDI simultaneously without mediating factors (model 2), those in the second highest quintile of healthy plant food consumption had a significantly lower risk of hospitalization with any infection (11% (HR 0.89 [95% CI: 0.79, 0.99])) compared to those in the lowest quintile (Table S7). Those in the highest quintile of healthy plant food consumption did not have a statistically significant difference in risk of hospitalization with infection (HR 0.89 [95% CI: 0.78, 1.01]), although the overall trend for healthy plant food consumption revealed a lower risk of hospitalization with infection with greater healthy plant food consumption (*p* = 0.039). However, after adjustment for BMI and diabetes, those in the highest quintile of healthy plant food consumption had a 14% (HR 0.86 [(95% CI: 0.76, 0.97]) lower risk of hospitalization with infection, and the trend remained (*p* = 0.006). For animal food consumption, those in the highest quintile had a 28% (HR 1.28 [95% CI: 1.11, 1.48]) greater risk of hospitalization with infection compared to those in the lowest quintile (*p* for trend < 0.001), but this association was no longer significant in the fully adjusted model, HR 1.10 (95% CI: 0.95, 1.27) (*p* for trend = 0.085). There were no statistically significant associations with hospitalization with any infection seen with less healthy plant food consumption. With regards to food groups, higher intake of refined grains was associated with increased risk of hospitalization with any infection across models 2 and 3 (Table S8). Inconsistent trends were seen with other food groups and risks of hospitalization.

## 4. Discussion

In this analysis of a community-based cohort of 11,955 US adults at baseline, a healthy plant-based diet, characterized by higher intake of whole grains, fruits, vegetables, nuts, legumes, tea, and coffee, was associated with lower risk of incident, severe respiratory infections (pneumonia and influenza) and overall severe infection requiring hospitalization. A less healthy plant-based diet, characterized by higher intakes of refined grains, potatoes, fruit juices, sugar-sweetened beverages, and sweets and desserts, was not associated with greater risk of incident, severe respiratory infections and overall severe infections requiring hospitalization. An overall plant-based diet, i.e., a diet higher in plant foods in general and lower in animal foods, was associated with a lower risk of hospitalization for any infections, but not specifically respiratory infections.

Our study is one of the few studies examining patterns of plant-based foods consumption and their association with infections, particularly respiratory infection, among the general population. The vegetarian diet, and diets generally high in plant foods, have been associated with modulation of immune and inflammatory responses, likely due to key micronutrients found in plant foods that are necessary for proper immune function [2,16]. In a study of Spanish children whose families enrolled in a nutritional education program, adoption of a Mediterranean diet, high in vegetable, fruit, and nut intake, was associated with a lower rate of upper respiratory tract infections and inflammatory complications [17]. Since the onset of the COVID-19 pandemic in early 2020, many have speculated on modifiable risk factors that may influence the risk of infection. Diet patterns are of particular interest, given close links to obesity, which has been shown to be a significant risk factor for adverse outcomes in those with COVID-19 [18]. It has been postulated that the low incidence of COVID-19 in places such as Sub-Saharan Africa may be partially driven by diets low in refined and processed foods, and high in plant foods [19]. This association of a lower risk of COVID-19 infection and severity with higher intakes of healthy plant foods was noted by Merino et al., in a study of adults in the United States and the United Kingdom [9]. Similarly, in a study by Kim et al., healthcare workers who reported following plant-based diets were associated with 73% lower odds of being infected with the COVID-19 infection [10].

While we observed an association of diets high in healthy plant foods and lower risk of respiratory infections, we did not see associations with diets high in any plant-based foods. This may have been driven by a few factors. In the PDI, all plant foods, regardless of their association with other disease risk, received positive scores. When looking at the components of the PDI, less healthy plant foods were not associated with a risk of respiratory infections. Given that these less healthy plant foods receive equal scores with healthier plant foods may have attenuated the impact of healthy plant foods, making associations with the overall PDI insignificant. Furthermore, the classification of certain plant foods, such as potatoes (one of the most commonly consumed plant foods) classified as unhealthy may be problematic, as there is mixed evidence of the association of potato consumption with cardiovascular disease and diabetes with some studies showing beneficial effects and others detrimental effects [20,21,22]. Classifying potatoes as an unhealthy plant food may have attenuated the association of HPDI and infections.

Obesity as a risk factor for infection and poor infectious outcomes has been highlighted during the COVID-19 pandemic [23,24,25]. Obesity is associated with a pro-inflammatory state with adipocytes releasing and signaling the release of many pro-inflammatory cytokines such as CRP and IL-6 [26]. Diabetes is strongly associated with obesity and is also associated with a pro-inflammatory state. It has been long associated with increased risk of infection and poor infectious outcomes [27,28,29]. Given that dietary patterns strongly contribute to the development of diabetes and obesity [30], and the association of obesity and diabetes with inflammation and worsened COVID-19 outcomes, we explored obesity and diabetes as mediators of the association of diet types and respiratory infections. In our analyses, inclusion of obesity and diabetes did not significantly change the associations of our diet indices and the risk of hospitalization with infection. However, when examining the score components (healthy plant foods, less healthy plant foods, and animal foods), those in the highest quintiles of less healthy plant food and animal consumption had an increased risk of hospitalization with respiratory and all-cause infections, which was no longer significant with the addition of BMI and diabetes in the models. This suggests that some of the association of less healthy plant food and animal food consumption may have been mediated through BMI and diabetes. The inclusion of BMI and diabetes strengthened the association of high healthy plant food consumption and decreased the risk of hospitalization with respiratory and all-cause infection. These differences suggest that certain types of foods may modulate the pro-inflammatory states of obesity and diabetes, which aligns with previous studies showing the importance of certain micronutrients in proper immune system function [3,31].

There are several important limitations to our study. Dietary intake was self-reported, which is prone to measurement error and recall bias. The FFQs used to determine dietary intake, while well-validated [15], may have misclassified some plant foods, and likely do not fully capture individuals’ complete dietary intake, as many food items, particularly ethnic foods, are not included. Furthermore, with regard to the outcome, some infections, particularly mild or moderate infections, were likely missed as only hospitalization data were used. With the current COVID-19 pandemic, there is a greater focus on severe infections requiring hospitalizations, but moderate infections that might not have led to hospitalization may still cause significant illness that impacts one’s quality of life. Additionally, we do not include any COVID-19 data in these analyses, but our analyses may have important implications for the COVID-19 pandemic. Lastly, due to the observational nature of this study, there is likely residual confounding not accounted for despite our robust adjustment.

Our study adds to the literature on plant-based diets and infection risk by using a community-based, national cohort of adults. We further characterize how types of plant-based diets and the consumption of healthy and less healthy plant foods and animal foods are associated with infection risk. These findings may have important clinical implications and further add to the body of evidence that supports improved infectious outcomes with healthy plant-based diets. Public health initiatives that aim to improve the diet quality of individuals may be especially important for those at risk of poor infectious outcomes, such as those with a low socioeconomic status, obesity, and diabetes. Dietary interventions are needed to reduce the risk of hospitalizations due to infections, and to complement effective evidence-based public health initiatives, such as vaccinations.

## 5. Conclusions

In conclusion, a healthy plant-based diet is associated with a lower risk of respiratory infections and infection-related hospitalizations. These findings reinforce the importance of public health messaging and policies surrounding healthy eating.

## Figures and Tables

**Table 1:** Participant Characteristics by quintiles of plant-based diet index, ARIC visit 1 (1987–1989) and visit 3 (1993–1995).

Characteristics	Quintile 1 (n = 2129)	Quintile 2 (n= 2554)	Quintile 3 (n = 2384)	Quintile 4 (n = 2759)	Quintile 5 (n = 2129)
Median score (range)	43(18–45)	48 (46–49)	51 (50–52)	54 (53–56)	59 (57–72)
Female, %	44.0	53.8	61.1	61.6	59.0
Mean age (SD)	59.8 (5.6)	59.9 (5.7)	59.8 (5.7)	60.2 (5.7)	60.2 (5.7)
Race, %					
White	67.7	74.5	77.9	82.2	87.0
Black	32.1	25.1	21.9	17.5	12.6
BMI, mean (SD)	29.1 (5.6)	29.0 (5.7)	28.4 (5.4)	28.3 (5.5)	27.7 (5.3)
Current smoker, %	24.5	19.3	17.4	13.5	12.5
Alcohol intake in g/week, mean (SD)	68.5 (159.2)	47.7 (125.5)	36.1 (112.6)	30.6 (74.4)	29.1 (69.3)
Chronic lung disease, %					
Asthma	4.6	4.5	4.9	4.6	5.0
COPD	5.0	5.3	4.6	3.7	4.1
Diabetes mellitus, %	16.9	16.0	15.9	14.1	13.0
Income, %					
<$ 50,000	33.9	33.9	31.3	29.0	24.8
$ 50,000–75,000	36.0	35.0	36.4	35.9	36.7
>$ 75,000	30.1	31.1	32.3	35.1	38.5
Education, %					
Less than HS	26.4	21.3	19.00	17.5	14.0
HS graduate	40.4	41.9	43.0	42.7	41.4
Some college or above	33.1	36.8	38.0	39.8	44.6
Food and nutrient intakes, mean (SD)					
Total energy (kcal)	1753.2 (649.1)	1565.3 (598.5)	1507.4 (562.3)	1554.5 (565.3)	1703.1 (571.3)
Healthy plant foods (servings/day)	5.9 (3.3)	6.6 (3.2)	7.3 (3.3)	8.2 (3.3)	10.0 (3.7)
Less healthy plant foods (servings/day)	4.6 (2.5)	4.7 (2.6)	4.8 (2.5)	5.2 (2.5)	6.1 (2.7)
Animal foods (servings/day)	5.5 (2.3)	4.3 (1.8)	3.8 (1.7)	3.5 (1.6)	3.2 (1.5)
Vegetables and fruit (servings/day)	2.8 (2.0)	3.1 (2.1)	3.4 (2.0)	3.9 (2.1)	4.7 (2.3)
Meat (servings/day)	2.1 (1.0)	1.6 (0.9)	1.4 (0.8)	1.3 (0.7)	1.2 (0.7)
Fish (servings/day)	0.35 (0.4)	0.29 (0.3)	0.28 (0.3)	0.28 (0.3)	0.26 (0.3)
Dairy (servings/day)	2.1 (1.7)	1.7 (1.3)	1.6 (1.2)	1.5 (1.1)	1.4 (1.1)
Fiber (g)	14.4 (7.6)	15.4 (7.4)	16.7 (7.6)	18.8 (7.6)	23.1 (9.2)
Vitamin A (IU)	8486.4 (6807.4)	9044.8 (8362.4)	9847.1 (8255.9)	10,923.4 (8355.6)	13,067.6 (10,183.3)
Vitamin C (mg)	108.0 (88.4)	113.8 (87.2)	126.6 (86.4)	142.4 (89.0)	169.7 (91.3)
Folate (mcg)	226.1 (114.3)	229.2 (114.3)	247.1 (119.2)	272.3 (117.5)	320.1 (129.8)
Vitamin B12 (mcg)	8.3 (4.5)	6.8 (3.8)	6.3 (3.7)	5.8 (3.6)	5.5 (3.5)
Iron (mg)	11.3 (4.8)	10.9 (5.0)	11.1 (5.2)	11.9 (5.4)	13.4 (5.9)
Calcium (mg)	756.6 (485.5)	653.3 (390.3)	632.7 (358.4)	644.3 (342.1)	668.8 (328.1)
Magnesium (mg)	249.6 (101.5)	238.0 (93.6)	241.2 (90.7)	257.6 (90.7)	292.3 (97.6)
Potassium (mg)	2622.9 (1072.6)	2502.9 (962.1)	2564.9 (947.8)	2735.4 (950.7)	3086.2 (975.3)
Zinc (mg)	12.1 (5.2)	10.4 (4.5)	9.9 (4.3)	9.9 (4.1)	10.2 (4.2)
Protein (g)	86.6 (35.2)	74.3 (29.4)	70.6 (27.8)	70.1 (27.8)	72.2 (27.3
Carbs (g)	185.9 (84.7)	184.7 (82.6)	188.9 (79.2)	204.7 (78.3)	239.2 (83.1)
Fat (g)					
Animal	50.1 (21.7)	38.3 (17.3)	33.4 (15.2)	31.4 (15.3)	29.1 (14.6)
Vegetable	19.6 (11.9)	19.6 (12.2)	19.3 (12.0)	20.6 (12.3)	24.7 (13.1)

**Table 2:** Risk of hospitalization for respiratory infections by quintiles of diet indices.

Diet Type	Quintile	HR and 95% CI			
		Unadjusted	Model 1 *	Model 2 ^#^	Model 3 ^&^
PDI	Quintile 1	1 (ref)	1 (ref)	1 (ref)	1 (ref)
	Quintile 2	0.86 (0.76, 0.97)	0.89 (0.78, 1.00)	0.94 (0.82, 1.01)	0.94 (0.82, 1.08)
	Quintile 3	0.85 (0.75, 0.97)	0.89 (0.78, 1.00)	0.97 (0.85, 1.12)	0.98 (0.86,1.13)
	Quintile 4	0.77 (0.69,0.88)	0.78 (0.68, 0.89)	0.88 (0.77, 1.01)	0.91 (0.80, 1.04)
	Quintile 5	0.78 (0.69, 0.89)	0.78 (0.70, 0.83)	0.90 (0.78, 1.04)	0.92 (0.80, 1.07)
	Trend *p* value	<0.001	<0.001	0.099	0.217
HPDI	Quintile 1	1 (ref)	1 (ref)	1 (ref)	1 (ref)
	Quintile 2	0.95 (0.83, 1.07)	0.95 (0.84, 1.08)	0.97 (0.85, 1.12)	0.97 (0.85, 1.12)
	Quintile 3	0.94 (0.83, 1.06)	0.90 (0.80, 1.02)	0.95 (0.83, 1.08)	0.97 (0.84, 1.11)
	Quintile 4	0.94 (0.82, 1.06)	0.87 (0.77, 0.99)	0.93 (0.81, 1.08)	0.95 (0.82, 1.10)
	Quintile 5	0.82 (0.73, 0.93)	0.74 (0.65, 0.84)	0.84 (0.73, 0.97)	0.86 (0.75, 0.99)
	Trend *p* value	0.003	<0.001	0.012	0.037
UPDI	Quintile 1	1 (ref)	1 (ref)	1 (ref)	1 (ref)
	Quintile 2	0.98 (0.88, 1.08)	0.99 (0.89, 1.10)	0.98 (0.88, 1.01)	1.00 (0.90, 1.12)
	Quintile 3	0.88 (0.78, 0.98)	0.90 (0.80, 1.00)	0.90 (0.79, 1.01)	0.91 (0.80, 1.02)
	Quintile 4	1.01 (0.92, 1.12)	0.98 (0.90, 1.10)	1.00 (0.89, 1.11)	1.01 (0.90, 1.12)
	Quintile 5	1.01 (0.91, 1.12)	1.01 (0.90, 1.13)	1.00 (0.88, 1.12)	1.03 (0.91, 1.16)
	Trend *p* value	0.29	0.58	0.85	0.57

* Adjusted for age, gender, race/center, total energy intake. ^#^ Adjusted for age, gender, race/center, total energy intake, tobacco use, alcohol use, education, income, asthma, COPD. ^&^ Adjusted for age, gender, race/center, total energy intake, tobacco use, alcohol use, education, income, asthma, COPD, BMI, diabetes. We assessed linearity (trend *p* value) by assigning the median value within each quintile of plant-based diet scores.

**Table 3:** Risk of hospitalization with any infection by quintiles of diet indices.

Diet Type	Quintile	HR and 95% CI			
		Unadjusted	Model 1 *	Model 2 ^#^	Model 3 ^&^
PDI	Quintile 1	1 (Ref)	1 (Ref)	1 (Ref)	1 (Ref)
	Quintile 2	0.87 (0.80, 0.96)	0.88 (0.80, 0.97)	0.93 (0.84, 1.3)	0.94 (0.84, 1.04)
	Quintile 3	0.85 (0.77, 0.94)	0.86 (0.78, 0.95)	0.92 (0.82, 1.02)	0.93 (0.84, 1.04)
	Quintile 4	0.83(0.76, 0.92)	0.83 (0.75, 0.91)	0.89 (0.81, 0.99)	0.92 (0.83, 1.02)
	Quintile 5	0.77 (0.70, 0.85)	0.76 (0.68, 0.84)	0.82 (0.74, 0.92)	0.85 (0.76, 0.96)
	Trend *p* value	<0.001	<0.001	<0.001	0.009
HPDI	Quintile 1	1 (Ref)	1 (Ref)	1 (Ref)	1 (Ref)
	Quintile 2	0.99 (0.90, 1.09)	0.98 (0.89, 1.08)	0.99 (0.89, 1.10)	0.98 (0.88, 1.09)
	Quintile 3	0.97 (0.88, 1.07)	0.92 (0.84, 1.02)	0.95 (0.85, 1.05)	0.97 (0.87, 1.07)
	Quintile 4	0.93 (0.84, 1.03)	0.86 (0.78, 0.95)	0.91 (0.81, 1.01)	0.92 (0.83, 1.03)
	Quintile 5	0.87 (0.79, 0.95)	0.78 (0.70, 0.86)	0.84 (0.76, 0.94)	0.87 (0.78, 0.97)
	Trend *p* value	0.001	<0.001	<0.001	0.006
UPDI	Quintile 1	1 (Ref)	1 (Ref)	1 (Ref)	1 (Ref)
	Quintile 2	0.98 (0.91, 1.06)	0.98 (0.91, 1.06)	0.98 (0.90, 1.07)	1.00 (0.92, 1.09)
	Quintile 3	0.96 (0.89, 1.05)	0.97 (0.89, 1.05)	0.96 (0.87, 1.05)	0.98 (0.90, 1.08)
	Quintile 4	0.98 (0.90, 1.05)	0.95 (0.88, 1.03)	0.97 (0.89, 1.05)	0.98 (0.90, 1.06)
	Quintile 5	1.00 (0.92, 1.09)	1.00 (0.91, 1.09)	0.98 (0.90, 1.08)	1.03 (0.94, 1.13)
	Trend *p* value	0.57	0.80	0.79	0.35

* Adjusted for age, gender, race/center, total energy intake. ^#^ Adjusted for age, gender, race/center, total energy intake, tobacco use, alcohol use, education, income, asthma, COPD. ^&^ Adjusted for age, gender, race/center, total energy intake, tobacco use, alcohol use, education, income, asthma, COPD, BMI, diabetes. We assessed linearity (trend *p* value) by assigning the median value within each quintile of plant-based diet scores.

## Data Availability

Data described in the manuscript, code book, and analytic code are available upon request to and at the discretion of the ARIC Data Coordinating Center.

## Supplementary Materials

The following supporting information can be downloaded at: https://www.mdpi.com/article/10.3390/nu15194265/s1, Supplementary Table S1. Unadjusted incidence rates of hospitalizations with respiratory infections by quintiles of diet indices. Supplementary Table S2. Risk of hospitalization with respiratory infections by quintiles of components of diet indices. Supplementary Table S3. Unadjusted incidence rates of hospitalizations with any infections by quintiles of diet indices. Supplementary Table S4. Risk of hospitalization with infections of any cause by quintiles of components of diet indices. Supplementary Table S5. Participant characteristics by quintiles of healthy plant-based diet index (HPDI). Supplementary Table S6. Participant characteristics by quintiles of unhealthy plant-based diet index (UPDI). Supplementary Table S7. Risk of hospitalization with respiratory infection by servings/day of food groups. Supplementary Table S8. Risk of hospitalization with infection of any cause by servings/day of food groups. Supplementary Figure S1. Participant flow chart. Supplementary Figure S2. Cumulative incidence of respiratory infections by quintiles of plant-based diet index. Supplementary Figure S3. Cumulative incidence of respiratory infections by quintiles of healthy plant-based diet index. Supplementary Figure S4. Cumulative incidence of respiratory infections by quintiles of unhealthy plant-based diet index.
