# Supplementary material for: Plant-Based Diets and Risk of Hospitalization with Respiratory Infection: Results from the Atherosclerosis Risk in Communities (ARIC) Study
Source: Nutrients. 2023 Oct 5;15(19):4265. doi: 10.3390/nu15194265 (PMC10574784; doi:10.3390/nu15194265)
Supplement: Supplementary file 1 [file nutrients-15-04265-s001.zip › nutrients-2600471-supplementary.pdf]

Supplement Material

Supplemental Table S1. Participant Characteristics by Quintiles of Healthy Plant-based Diet Index (HPDI)

Supplemental Table S2. Participant Characteristics by Quintiles of Unhealthy Plant-based Diet Index (UPDI)

Supplemental Table S3. Unadjusted Incidence Rates of Hospitalizations with respiratory infections by Quintiles of Diet Indices

Supplemental Table S4. Risk of Hospitalization with Respiratory Infections by Quintiles of Components of Diet Indices

Supplemental Table S5. Risk of Hospitalization with Respiratory Infection by Servings/day of Food Groups

Supplemental Table S6. Unadjusted Incidence Rates of Hospitalizations with Any infections by Quintiles of Diet Indices

Supplemental Table S7. Risk of Hospitalization with Infections of Any Cause by Quintiles of Components of Diet Indices

Supplemental Table S8. Risk of Hospitalization with Any Infection by Servings/day of Food Groups

Supplemental Figure S1. Participant Flow Chart

Supplemental Figure S2. Cumulative Incidence of Respiratory Infections by Quintiles of Plant-based Diet Index

Supplemental Figure S3. Cumulative Incidence of Respiratory Infections by Quintiles of Healthy Plant-based Diet Index

Supplemental Figure S4. Cumulative Incidence of Respiratory Infections by Quintiles of Unhealthy Plant-based Diet Index

Table S1. Participant Characteristics by Quintiles of Healthy Plant-based Diet Index.

Characteristics	Quintile 1 (n=2135)	Quintile 2 (n=2396)	Quintile 3 (n=2645)	Quintile 4 (n=2163)	Quintile 5 (n=2616)
Median score (range)	42 (26-44)	47 (45-48)	51 (49-52)	54 (53-56)	60 (57-77)
Female, %	48.7	52.8	56.3	60.5	61.8
Mean age (SD)	59.1 (5.6)	59.5 (5.7)	60.1 (5.7)	60.2 (5.7)	60.7 (5.6)
Race, %<br>White<br>Black	 69.1<br>30.8	 75.2<br>24.6	 79.9<br>19.7	 80.7<br>19.0	 83.6<br>16.1
BMI, mean (SD)	29.3 (5.7)	29.0 (5.7)	28.4 (5.4)	28.2 (5.3)	27.7 (5.3)
Current smoker, %	19.6	19.4	17.2	16.6	14.1
Alcohol intake in g/week, mean (SD)	36.9 (110.3)	45.9 (132.3)	40.7 (100.7)	41.5 (108.2)	43.6 (110.8)
Chronic lung disease, %<br>Asthma<br>COPD	4.1<br>5.0	4.8<br>3.8	5.1<br>4.7	4.9<br>4.4	4.6<br>4.8
Diabetes mellitus	16.7	15.2	14.8	16.2	13.4
Income, %<br><50,000<br>50,000-75,000<br>>75,000	 34.8<br>33.8<br>31.4	 32.5<br>35.4<br>32.1	 29.8<br>37.5<br>32.7	 31.2<br>34.8<br>34.0	 25.8<br>37.6<br>36.6
Education, %<br>Less than HS<br>HS graduate<br>Some college and above	 27.5<br>41.1<br>31.4	 21.3<br>43.5<br>35.1	 19.2<br>43.0<br>37.8	 16.6<br>42.7<br>40.6	 14.5<br>39.5<br>46.0
Food and nutrient intakes, mean (SD)					
Total energy (kcal)	1528.3 (608.4)	1482.4 (593.4)	1543.1 (579.8)	1649.2 (585.5)	1824.9 (549.2)
Healthy plant foods (servings/day)	4.6 (2.3)	5.9 (2.4)	7.2 (2.6)	8.6 (2.8)	11.2 (3.5)
Less healthy plant foods (servings/day)	5.9 (2.8)	5.2 (2.6)	5.0 (2.6)	4.8 (2.6)	4.6 (2.4)
Animal foods (servings/day)	4.4 (2.0)	3.9 (1.9)	3.9 (1.9)	3.9 (2.0)	4.1 (2.0)
Vegetables and fruit (servings/day)	2.0 (1.2)	2.6 (1.4)	3.3 (1.5)	4.0 (1.9)	5.6 (2.6)
Meat (servings/day)	1.7 (0.9)	1.5 (0.9)	1.4 (0.9)	1.5 (0.9)	1.4 (0.9)
Fish (servings/day)	0.3 (0.3)	0.3 (0.3)	0.3 (0.3)	0.3 (0.3)	0.3 (0.4)
Dairy (servings/day)	1.5 (1.2)	1.5 (1.2)	1.6 (1.2)	1.7 (1.4)	2.0 (1.5)
Fiber (g)	12.5 (5.7)	14.1 (6.0)	16.6 (6.8)	19.3 (7.6)	24.7 (9.3)

Vitamin A (IU)	6587.1 (4750.9	7779.8 (5730.1)	9451.8 (6537.4)	11250.6 (7837.2)	15507.6 (12205.1)
Vitamin C (mg)	109.1 (77.8)	110.6 (77.5)	129.4 (87.4)	139.6 (94.4)	166.0 (101.3)
Folate (mcg)	208.0 (101.0)	218.1 (99.4)	249.0 (108.5)	275.0 (123.0)	331.1 (137.1)
Vitamin B12 (mcg)	6.7 (4.3)	6.4 (3.9)	6.4 (3.9)	6.5 (3.9)	6.6(4.1)
Iron (mg)	10.3 (4.7)	10.5 (4.9)	11.2 (5.2)	12.3 (5.4)	13.9 (5.7)
Calcium (mg)	586.8 (341.2)	583.4 (335.9)	636.5 (353.9)	696.2 (398.0)	821.6
Magnesium (mg)	206.0 (78.3)	218.9 (79.7)	244.3 (82.2)	271.3 (88.8)	324.9 (99.7)
Potassium (mg)	2184.8 (827.4)	2314.9 (828.9)	2588.6 (844.2)	2860.6 (926.6)	3425.9 (1017.6)
Zinc (mg)	9.8 (4.3)	9.7 (4.5)	10.1 (4.3)	10.7 (4.5)	11.8 (4.6)
Protein (g)	68.8 (27.6)	67.9 (28.2)	71.1 (27.7)	76.9 (29.9)	86.4 (32.4)
Carbs (g)	117.7 (80.5)	176.7 (76.8)	191.0 (79.1)	208.8 (81.3)	242.5 (82.05)
Fat (g)<br>Animal<br>Vegetable	 39.9 (18.6)<br>20.7 (12.0)	 36.0 (18.6)<br>19.6 (12.2)	 35.0 (18.3)<br>19.8 (11.9)	 35.5 (18.3)<br>20.9 (12.3)	 35.3 (17.9)<br>22.6 (13.5)

Table S2. Participant Characteristics by Quintiles of Unhealthy Plant-based Diet Index.

Characteristics	Quintile 1 (n=2348)	Quintile 2 (n=2748	Quintile 3 (n=2269)	Quintile 4 (n=2324)	Quintile 5 (n=2266)
Median score (range)	43 (20-45)	48 (46-49)	51 (50-52)	54 (53-56)	60 (57-76)
Female, %	61.6	60.1	57.6	52.5	48.2
Mean age (SD)	60.4 (5.7)	60.2 (5.6)	60.0 (5.7)	59.8 (5.7)	59.3 (5.7)
Race, %<br>White<br>Black	 79.3<br>20.4	 76.9<br>22.7	 77.4<br>22.3	 77.3<br>22.5	 79.2<br>20.7
BMI, mean (SD)	28.6 (5.6)	28.5 (5.4)	28.4 (5.7)	28.5 (5.5)	28.4 (5.5)
Current smoker, %	19.2	17.1	14.9	16.7	18.5
Alcohol intake in g/week, mean (SD)	36.1 (101.3)	39.3 (111.1)	38.2 (94.7)	44.3 (113.5)	52.0 (138.7)
Chronic lung disease, %<br>Asthma<br>COPD	4.3<br>4.7	4.5<br>4.3	5.5<br>4.5	4.8<br>4.3	4.3<br>4.9
Diabetes mellitus	19.9	15.2	15.9	12.9	11.8
Income, %<br><50,000<br>50,000-75,000<br>>75,000	 30.0<br>37.2<br>32.9	 31.6<br>36.1<br>32.4	 29.9<br>35.7<br>33.9	 29.9<br>35.7<br>34.4	 31.4<br>34.7<br>33.9
Education , %<br>Less than HS<br>HS graduate<br>Some college and above	 18.3<br>40.6<br>41.1	 18.5<br>41.7<br>39.8	 19.2<br>41.5<br>39.3	 20.1<br>42.5<br>37.5	 22.3<br>43.7<br>34.1
Food and nutrient intakes, mean (SD)					
Total energy intake (kcal)	1564.2 (570.8)	1493.7 (561.7)	1506.4 (556.4)	1606.1 (579.2)	1901.5 (619.6)
Healthy plant foods (servings/day)	9.7 (3.5)	8.1 (3.4)	7.3 (3.4)	6.8 (3.3)	6.0 (3.1)
Less healthy plant foods (servings/day)	3.3 (1.7)	4.1 (1.9)	4.7 (2.1)	5.6 (2.2)	7.8 (2.9)
Animal foods (servings/day)	4.5 (2.1)	3.9 (1.9)	3.8 (1.8)	3.8 (2.0)	4.0 (1.9)
Vegetables and fruit (servings/day)	4.5 (2.2)	3.8 (2.3)	3.5 (2.2)	3.2 (2.0)	2.9 (1.9)
Meat (servings/day)	1.6 (0.9)	1.5 (0.9)	1.4 (0.8)	1.5 (0.9)	1.6 (0.9)
Fish (servings/day)	0.4 (0.4)	0.3 (0.3)	0.3 (0.3)	0.2 (0.3)	0.2 (0.3)
Dairy (servings/day)	1.9 (1.3)	1.6(1.2)	1.6(1.2)	1.6(1.4)	1.6 (1.4)
Fiber (g)	19.7 (8.5)	17.7 (8.5)	16.9 (8.5)	16.6 (8.1)	17.1 (8.1)

Vitamin A (IU)	13136(9405)	10965 (9285)	9797 (7488)	8918 (7488)	8239 (6898)
Vitamin C (mg)	127.0 (78.2)	125.8 (83.7)	127.9 (88.5)	129.6 (87.9)	150.6 (112.4)
Folate (mcg)	273.6 (118.4)	253.8 (119.1)	250.6 (127.3)	248.6 (118.2)	264.4 (133.3)
Vitamin B12 (mcg)	7.3 (4.2)	6.4 (3.9)	6.2 (3.8)	6.1 (3.8)	6.5 (3.8)
Iron (mg)	11.9 (4.9)	11.2 (4.8)	11.2 (5.6)	11.5 (5.5)	12.6 (6.0)
Calcium (mg)	734.0 (392.0)	650.2 (363.8)	635.9 (376.9)	642.9 (391.8)	680.5 (394.3)
Magnesium (mg)	280.2 (97.0)	252.0 (95.4)	244.3 (96.8)	243.0 (93.9)	255.0 (94.4)
Potassium (mg)	2967.5 (1007.8)	2675.1 (996.3)	2589.7 (1001.6)	2566.2 (984.3)	2670.0 (955.6)
Zinc (mg)	11.0 (4.6)	10.1 (4.4)	9.9 (4.3)	10.2 (4.6)	11.1 (4.7)
Protein (g)	82.4 (32.4)	73.6 (29.8)	71.1 (29.2)	70.9 (29.5)	74.2 (27.9)
Carbs (g)	183.4 (72.5)	182.0 (75.6)	187.7 (78.2)	202.4 (79.1)	249.5 (94.0)
Fat (g)					
Animal	38.5 (19.5)	34.7 (18.3)	33.7 (16.8)	35.2 (18.3)	39.3 (18.2)
Vegetable	17.8 (10.4)	18.1 (10.6)	19.0 (11.2)	21.6 (12.5)	27.7 (14.7)

Table S3. Unadjusted Incidence Rates of Hospitalizations with Respiratory Infections by Quintiles of Diet Indices.

Incident Rates (per 1,000 PY) by Quintiles of Diet Indices	
PDI	
Quintiles	IR (95% CI)
1	12.96 (11.87, 14.16)
2	11.31 (10.39, 12.30)
3	11.19 (10.26, 12.21)
4	10.42 (9.58, 11.33)
5	10.64 (9.69, 11.68)
HPDI	
Quintiles	
1	12.02 (10.99, 13.15)
2	11.40 (10.45, 12.43)
3	11.58 (10.67, 12.56)
4	11.26 (10.28, 12.34)
5	10.09 (9.24, 11.01)
UPDI	
Quintiles	
1	11.87 (10.90, 12.94)
2	11.06 (10.19, 12.00)
3	10.31 (9.38, 11.32)
4	11.27 (10.32, 12.31)
5	11.66 (10.68, 12.74)

Table S4. Risk of Hospitalization with Respiratory Infections by Quintiles of Components of Diet Indices.

Diet type		HR and 95% CI			
	Quintile	Unadjusted	Model 1*	Model 2#	Model 3&
Healthy plant foods	Quintile 1<br>Quintile 2<br>Quintile 3<br>Quintile 4<br>Quintile 5	1 (Ref)<br>0.91 (0.80, 1.03)<br>0.96 (0.85,1.09)<br>0.91 (0.81, 1.03)<br>0.84 (0.74, 0.96)	1 (Ref)<br>0.88 (0.78, 1.00)<br>0.90 (0.80, 1.03)<br>0.88 (0.78, 1.01)<br>0.86 (0.75, 1.00)	1 (Ref)<br>0.93 (0.81, 1.07)<br>0.92 (0.80, 1.06)<br>0.91 (0.79, 1.06)<br>0.87 (0.74, 1.01)	1 (Ref)<br>0.94 (0.82, 1.08)<br>0.93 (0.81, 1.07)<br>0.91 (0.79, 1.05)<br>0.84 (0.71, 0.98)
	Trend p value	0.016	0.09	0.08	0.027
Less healthy plant foods	Quintile 1<br>Quintile 2<br>Quintile 3<br>Quintile 4<br>Quintile 5	1 (Ref)<br>1.02 (0.90 1.16)<br>1.01 (0.90, 1.16)<br>0.95 (0.83, 1.08)<br>1.18 (1.04, 1.34)	1 (Ref)<br>1.01 (0.89, 1.16)<br>1.02 (0.90, 1.16)<br>0.98 (0.85, 1.13)<br>1.26 (1.08, 1.48)	1 (Ref)<br>1.08 (0.94, 1.24)<br>1.04 (0.90, 1.20)<br>1.00 (0.86, 1.16)<br>1.24 (1.04, 1.48)	1 (Ref)<br>1.07 (0.93, 1.22)<br>1.03 (0.89, 1.18)<br>0.97 (0.83, 1.13)<br>1.19 (1.00, 1.42)
	Trend p value	0.016	0.006	0.048	0.13
Animal foods	Quintile 1<br>Quintile 2<br>Quintile 3<br>Quintile 4<br>Quintile 5	1 (Ref)<br>0.97 (0.85, 1.10)<br>1.04 (0.91, 1.18)<br>1.16 (1.02, 1.32)<br>1.28 (1.12, 1.46)	1 (Ref)<br>0.94 (0.83, 1.07)<br>1.01 (0.89, 1.16)<br>1.15 (1.00, 1.33)<br>1.30 (1.10, 1.54)	1 (Ref)<br>0.92 (0.80, 1.06)<br>0.98 (0.84, 1.13)<br>1.11 (0.95, 1.30)<br>1.21 (1.01, 1.46)	1 (Ref)<br>0.90 (0.78, 1.03)<br>0.92 (0.79, 1.06)<br>1.03 (0.88, 1.20)<br>1.07 (0.89, 1.29)
	Trend p value	<0.001	<0.001	0.01	0.23

*adjusted for age, gender, race-center, total energy intake

#adjusted for age, gender, race-center, total energy intake, tobacco use, alcohol use, education, income, asthma, COPD

&adjusted for age, gender, race-center, total energy intake, tobacco use, alcohol use, education, income, asthma, COPD, BMI, diabetes.

In these Cox regression models, we adjusted for healthy plant foods, less healthy plant foods, and animal foods simultaneously.

COPD, chronic obstructive pulmonary disease

Table S5. Risk of Hospitalization with Respiratory Infection by Servings/day of Food Groups.

Food groups	HR 95% Confidence Limits			
	**Unadjusted**	**Model 1***	**Model 2#**	**Model 3&**
**Coffee and Tea**	1.00 (0.98, 1.02)	1.01 (0.99, 1.03)	0.98 (0.96, 1.00)	0.98 (0.96, 1.00)
**Whole grains**	0.92 (0.88, 0.96)	0.93 (0.89, 0.98)	0.97 (0.92, 1.02)	0.96 (0.91, 1.01)
**Fruits**	0.99 (0.96, 1.02)	0.99 (0.95, 1.02)	1.02 (0.98, 1.06)	1.00 (0.97, 1.04)
**Vegetables**	0.99 (0.95, 1.03)	0.99 (0.05, 1.03)	1.00 (0.95, 1.05)	0.99 (0.95, 1.04)
**Nuts**	1.01 (0.94, 1.10)	0.98 (0.90, 1.07)	1.00 (0.91, 1.09)	0.98 (0.89, 1.07)
**Legumes**	1.06 (0.98, 1.14)	1.02 (0.94, 1.11)	0.98 (0.90, 1.07)	0.98 (0.89, 1.07)
**Juice**	1.00 (0.94, 1.06)	0.99 (0.93, 1.06)	1.01 (0.94, 1.08)	1.00 (0.93, 1.07)
**Refined grains**	1.03 (1.00, 1.06)	1.01 (0.98, 1.06)	1.03 (1.00, 1.07)	1.03 (0.99, 1.06)
**Potatoes**	0.98 (0.87, 1.10)	1.03 (0.91, 1.16)	1.09 (0.96, 1.25)	1.07 (0.94, 1.23)
**Sugar sweetened beverages**	1.03 (1.00, 1.07)	1.09 (1.05, 1.13)	1.08 (1.04, 1.12)	1.05 (1.01, 1.09)
**Sweets**	0.98 (0.95, 1.02)	0.99 (0.94, 1.03)	0.99 (0.94, 1.04)	0.99 (0.94, 1.04)
**Animal fats**	0.98 (0.92, 1.04)	0.99 (0.93, 1.05)	0.96 (0.90, 1.02)	0.95 (0.89, 1.02)
**Dairy**	1.02 (0.99, 1.05)	1.02 (0.98, 1.06)	1.04 (0.99, 1.08)	1.01 (0.97, 1.06)
**Eggs**	1.32 (1.19,1.46)	1.27 (1.14, 1.41)	1.18 (1.04, 1.33)	1.13 (1.00, 1.28)
**Fish**	1.03 (0.91, 1.17)	1.10 (0.96, 1.26)	1.16 (1.01, 1.34)	1.10 (0.96, 1.27)
**Meat**	1.06 (1.00, 1.11)	1.08 (1.02, 1.16)	1.10 (1.02, 1.18)	1.03 (0.96, 1.12)
**Animal misc**	1.03 (0.92, 1.14)	1.02 (0.92, 1.13)	0.97 (0.86, 1.09)	0.96 (0.85, 1.08)

*adjusted for age, gender, race-center, total energy intake
#adjusted for age, gender, race-center, total energy intake, tobacco use, alcohol use, education, income, asthma, COPD
&adjusted for age, gender, race-center, total energy intake, tobacco use, alcohol use, education, income, asthma, COPD, BMI, diabetes.

Table S6. Unadjusted Incidence Rates of Hospitalizations with Any infections by Quintiles of Diet Indices.

Incident Rates (per 1,000 PY) by Quintiles of Diet Indices	
PDI	
Quintiles	IR (95% CI)
1	22.51 (21.01, 24.12)
2	20.42 (19.14, 21.78)
3	19.84 (18.56, 21.22)
4	19.66 (18.47, 20.92)
5	18.72 (17.41, 20.12)
HPDI	
Quintiles	
1	21.09 (19.67, 22.62)
2	20.76 (19.44, 22.17)
3	20.54 (19.29, 21.87)
4	19.75 (18.41, 21.20)
5	18.83 (17.64, 20.11)
UPDI	
Quintiles	
1	21.13 (19.78, 22.58)
2	19.99 (18.79, 21.27)
3	19.59 (18.27, 21.00)
4	19.79 (18.49, 21.18)
5	20.34 (18.99, 21.78)

Table S7. Risk of Hospitalization with Any Infections by Quintiles of Components of Diet Indices.

Diet type		HR and 95% CI			
	Quintile	Unadjusted	Model 1*	Model 2#	Model 3&
Healthy plant foods	Quintile 1	Ref	Ref	Ref	Ref
	Quintile 2	0.93 (0.85, 1.02)	0.91 (0.83, 1.00)	0.94 (0.85, 1.04)	0.95 (0.85, 1.05)
	Quintile 3	0.99 (0.90, 1.09)	0.95 (0.86, 1.04)	0.97 (0.87, 1.07)	0.97 (0.87, 1.08)
	Quintile 4	0.89 (0.80, 0.97)	0.87 (0.79, 0.97)	0.89 (0.79, 0.99)	0.89 (0.79, 0.99)
	Quintile 5	0.86 (0.78, 1.03)	0.90 (0.80, 1.00)	0.89 (0.79, 1.01)	0.86 (0.76, 0.97)
	Trend p value	0.002	0.049	0.039	0.006
Less healthy plant foods	Quintile 1	Ref	Ref	Ref	Ref
	Quintile 2	0.98 (0.89, 1.08)	1.00 (0.91, 1.11)	1.04 (0.93, 1.14)	1.03 (0.92, 1.14)
	Quintile 3	0.98 (0.89, 1.08)	1.01 (0.91, 1.11)	1.02 (0.92, 1.14)	1.02 (0.91, 1.13)
	Quintile 4	0.92 (0.83, 1.01)	0.97(0.88, 1.08)	0.97 (0.87, 1.09)	0.94 (0.84, 1.06)
	Quintile 5	1.07 (0.97,1.19)	1.20 (106, 1.36)	1.16 (1.01, 1.33)	1.11 (0.97, 1.27)
	Trend p value	0.19	0.007	0.093	0.30
Animal foods	Quintile 1	Ref	Ref	Ref	Ref
	Quintile 2	0.99 (0.90, 1.09)	0.99 (0.90, 1.09)	0.97 (0.87, 1.08)	0.93 (0.83, 1.03)
	Quintile 3	1.09 (0.99, 1.20)	1.10 (0.99, 1.22)	1.07 (0.96, 1.20)	0.98 (0.88, 1.10)
	Quintile 4	1.15 (1.04, 1.27)	1.17 (1.05, 1.31)	1.13 (1.00, 1.27)	1.04 (0.92, 1.17)
	Quintile 5	1.29 (1.16, 1.42)	1.37 (1.20, 1.56)	1.28 (1.11, 1.48)	1.10 (0.95, 1.27)
	Trend p value	<0.001	<0.001	<0.001	0.09

*adjusted for age, gender, race-center, total energy intake

#adjusted for age, gender, race-center, total energy intake, tobacco use, alcohol use, education, income, asthma, COPD

&adjusted for age, gender, race-center, total energy intake, tobacco use, alcohol use, education, income, asthma, COPD, BMI, diabetes.

In Cox regression models, we adjusted for healthy plant foods, less healthy plant foods, and animal foods simultaneously.

Table S8. Risk of Hospitalization with Any Infection by Servings/day of Food Groups.

Food groups	HR 95% Confidence Limits			
	Unadjusted	Model 1*	Model 2#	Model 3&
**Coffee and Tea**	1.00 (0.98, 1.01)	1.01 (1.00, 1.03)	0.99 (0.98,1.01)	0.99(0.98, 1.01)
**Whole grains**	0.94 (0.91, 0.97)	0.96 (0.93, 1.00)	0.98 (0.94, 1.02)	0.97 (0.93, 1.00)
**Fruits**	0.98 (0.95, 1.00)	0.98 (0.95, 1.01)	1.00 (0.97, 1.03)	0.98 (0.95, 1.01)
**Vegetables**	1.00 (0.96, 1.03)	1.00 (0.97, 1.03)	1.01 (0.97, 1.05)	1.00 (0.96, 1.04)
**Nuts**	1.02 (0.96, 1.08)	1.02 (0.96, 1.09)	1.04 (0.98, 1.12)	1.02 (0.96, 1.09)
**Legumes**	1.05 (1.00, 1.12)	1.03 (0.98, 1.10)	1.00 (0.93, 1.06)	0.99 (0.92, 1.06)
**Juice**	0.98 (0.94, 1.03)	1.00 (0.95, 1.05)	1.02 (0.96, 1.07)	1.01 (0.96, 1.07)
**Refined grains**	1.03 (1.01, 1.05)	1.03 (1.00, 1.05)	1.04 (1.01, 1.07)	1.03 (1.02, 1.06)
**Potatoes**	0.96 (0.87, 1.05)	1.05 (0.96, 1.16)	1.07 (0.96, 1.19)	1.04 (0.94, 1.16)
**Sugar sweetened beverages**	1.03 (1.00, 1.05)	1.08 (1.05, 1.11)	1.07 (1.04, 1.10)	1.03 (1.00, 1.06)
**Sweets**	0.97 (0.94, 1.00)	1.00 (0.96, 1.03)	0.98 (0.95, 1.02)	0.98 (0.95, 1.02)
**Animal fats**	1.03 (0.98, 1.07)	1.05 (1.01, 1.10)	1.04 (0.99, 1.09)	1.03 (0.98, 1.08)
**Dairy**	1.02 (1.00, 1.05)	1.04 (1.01, 1.08)	1.05 (1.01, 1.08)	1.02 (0.99, 0.99)
**Eggs**	1.23 (1.13, 1.34)	1.20 (1.10, 1.31)	1.10 (1.00, 1.21)	1.05 (0.95, 1.16)
**Fish**	1.03 (0.93, 1.14)	1.11 (1.01, 1.23)	1.14 (1.02, 1.28)	1.08 (0.96, 1.21)
**Meat**	1.05 (1.01, 1.09)	1.11 (1.05, 1.17)	1.12 (1.06, 1.19)	1.04 (0.98, 1.11)
**Animal misc**	1.02 (0.94, 1.11)	1.04 (0.95, 1.12)	0.99 (0.90, 1.08)	0.97 (0.88, 1.06)

*adjusted for age, gender, race-center, total energy intake
#adjusted for age, gender, race-center, total energy intake, tobacco use, alcohol use, education, income, asthma, COPD
&adjusted for age, gender, race-center, total energy intake, tobacco use, alcohol use, education, income, asthma, COPD, BMI, diabetes.

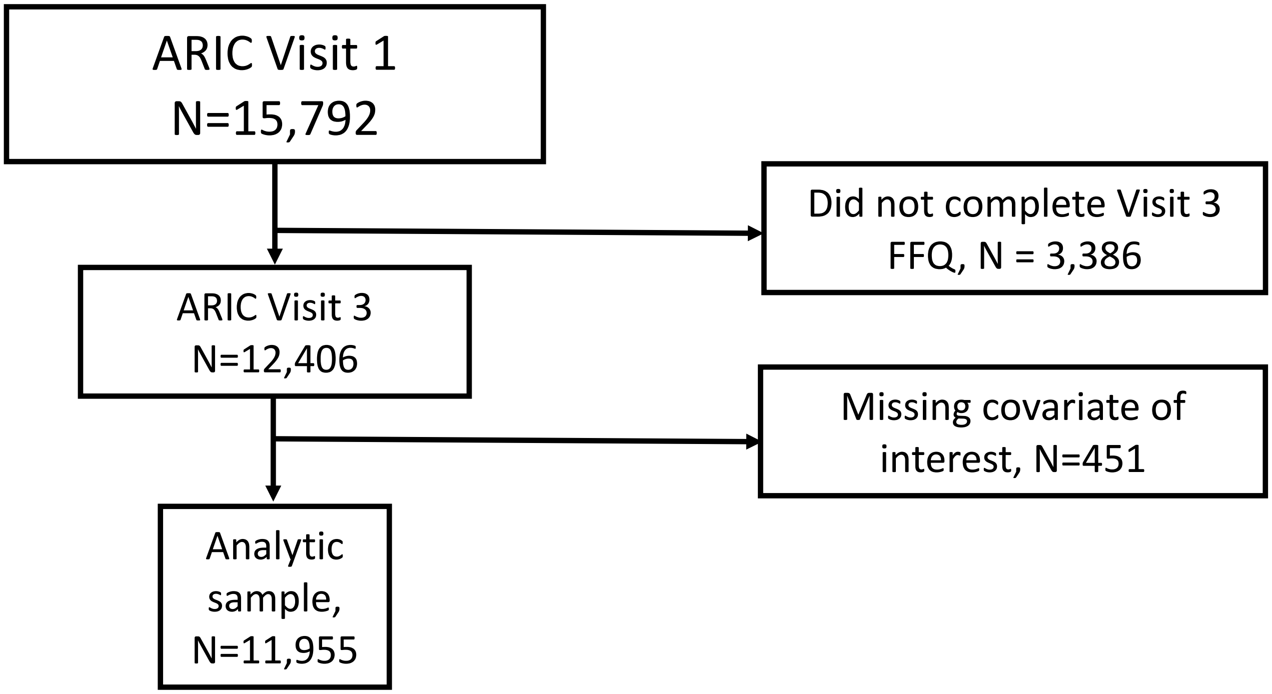

Figure S1. Participant Flow Chart.

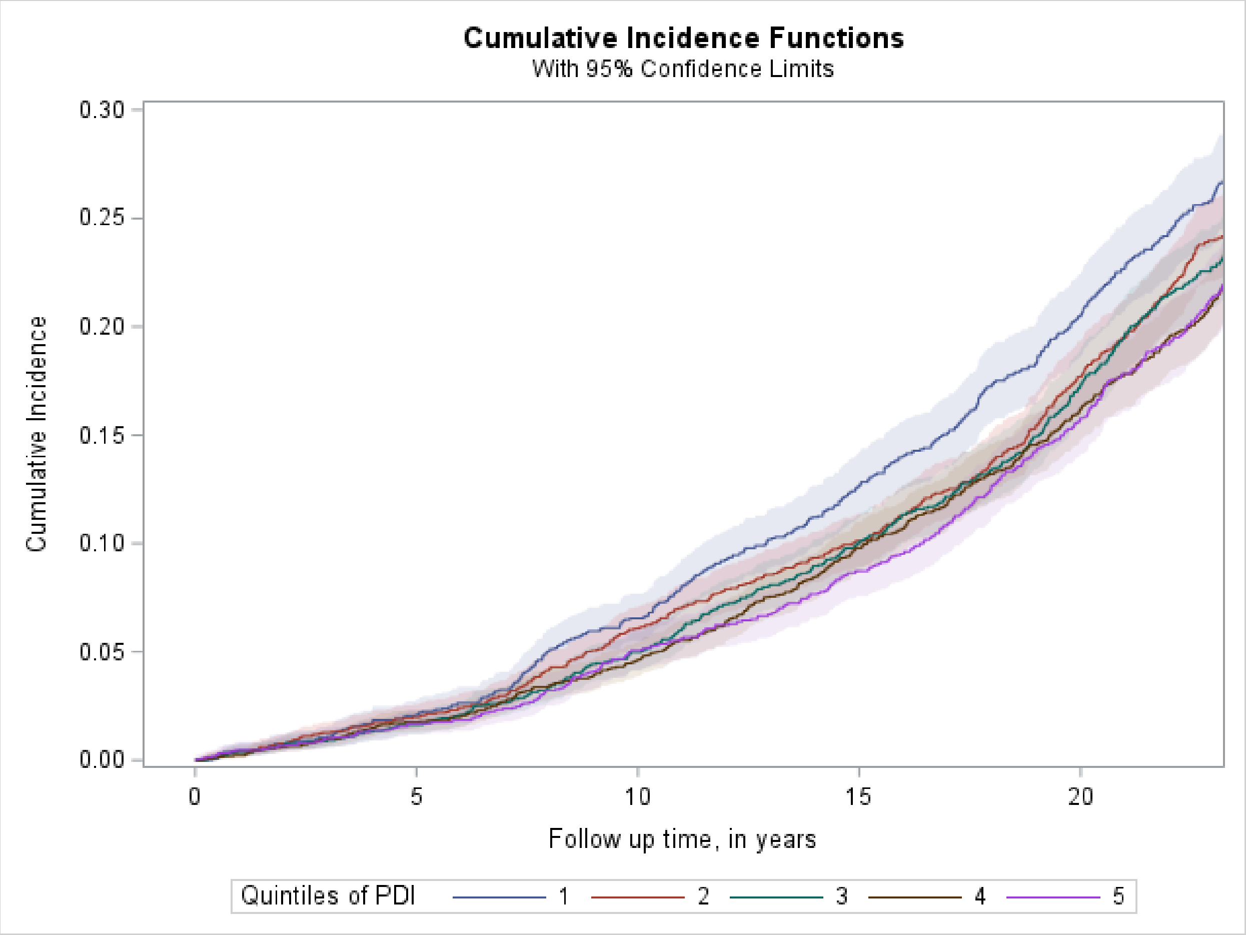

Figure S2. Cumulative Incidence of Respiratory Infections by Quintiles of Plant-based Diet Index.

This plot shows the cumulative incidence of respiratory infections by quintiles of the plant-based diet index with follow up time truncated after median follow up time. Shaded areas represent 95% confidence limits.

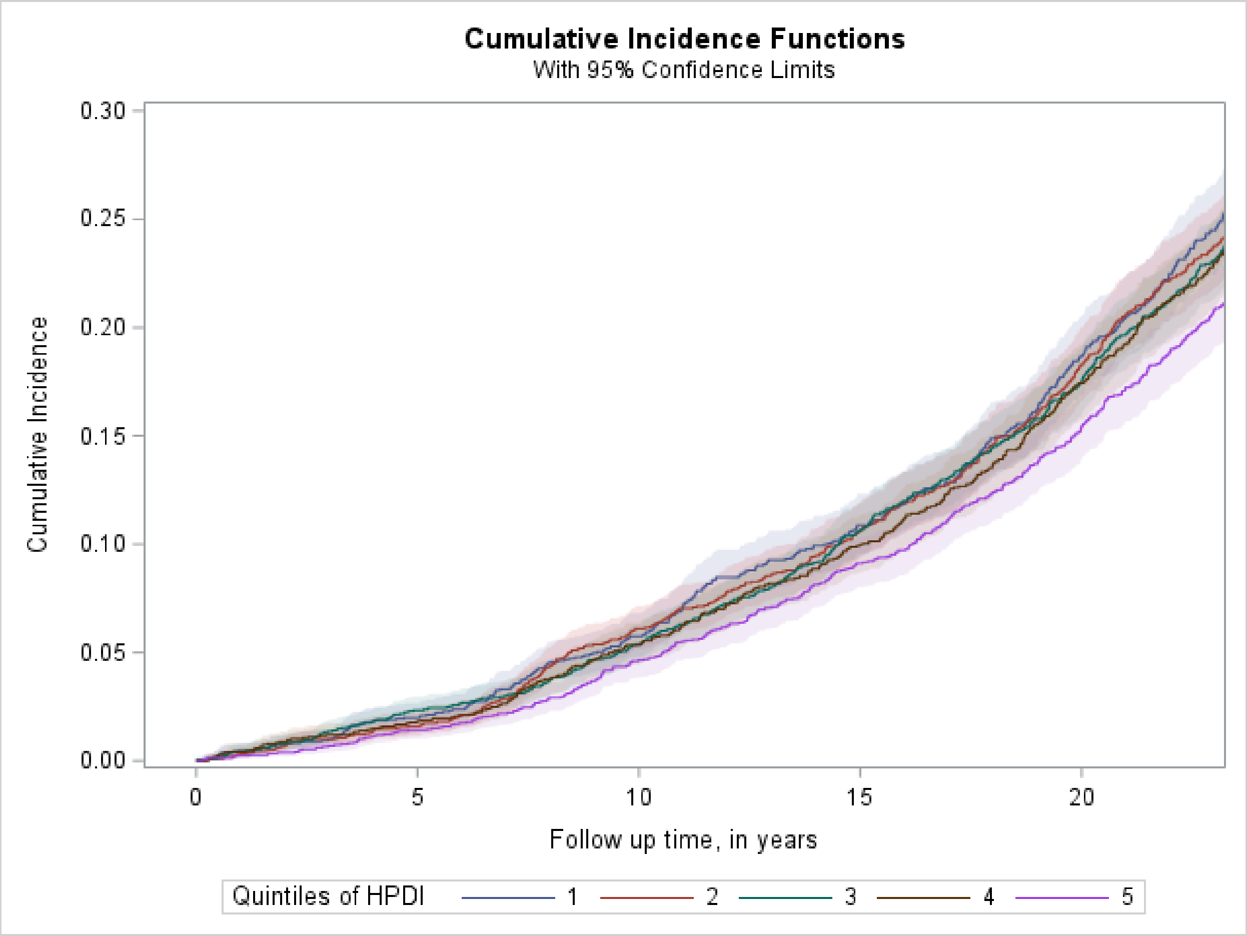

Figure S3. Cumulative Incidence of Respiratory Infections by Quintiles of Healthy Plant-based Diet Index.

This plot shows the cumulative incidence of respiratory infections by quintiles of the healthy plant-based diet index with follow up time truncated after median follow up time. Shaded areas represent 95% confidence limits.

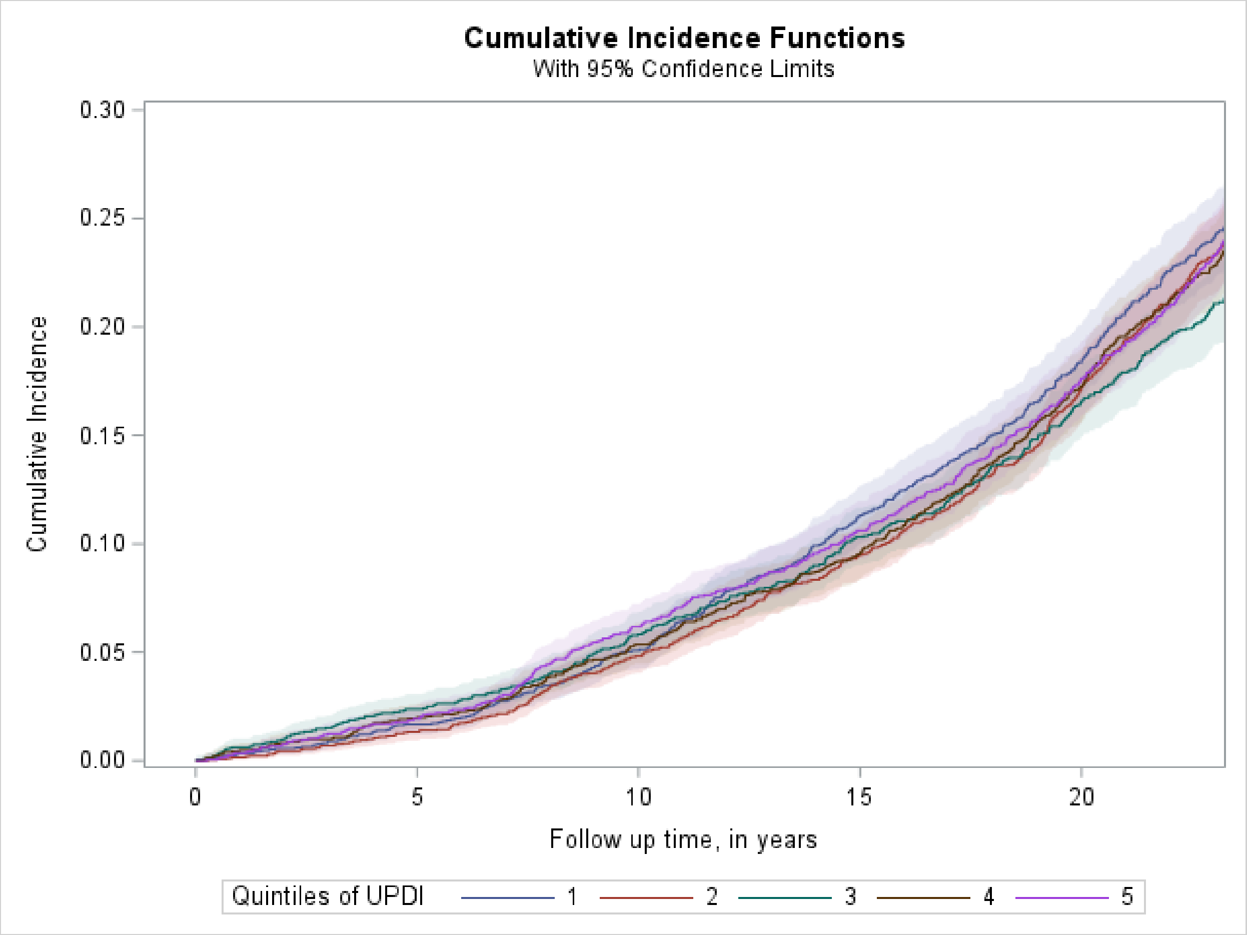

Figure S4. Cumulative Incidence of Respiratory Infections by Quintiles of Unhealthy Plant-based Diet Index.

This plot shows the cumulative incidence of respiratory infections by quintiles of the unhealthy plant-based diet index with follow up time truncated after median follow up time. Shaded areas represent 95% confidence limits.
